# Editorial: Toward a better understanding of the pathophysiology and clinical management of idiopathic normal pressure hydrocephalus

**DOI:** 10.3389/fneur.2022.1013720

**Published:** 2022-09-15

**Authors:** Kostadin L. Karagiozov, Ville Leinonen, Masakazu Miyajima, Madoka Nakajima

**Affiliations:** ^1^Department of Neurosurgery, Jikei University School of Medicine, Minato, Japan; ^2^Department of Neurosurgery, University of Kuopio, Kuopio, Finland; ^3^Department of Neurosurgery, Juntendo University, Bunkyo, Japan

**Keywords:** iNPH, pathophysiology, clinical management, shunting surgery, comorbidity

Considering the broad spectrum of unsolved problems in the understanding of iNPH, the authors contributing to this Research Topic have addressed very diverse research interests. This illustrates the significant deficiencies in research on this subject.

Although not strictly defined as clinically applied pathophysiology, two studies explore this subject. Jeppsson et al. explore related vascular pathology and Martinoni et al. examine some mechanisms in late-onset idiopathic aqueductal stenosis (LIAS), a condition similar to iNPH.

Jeppsson et al. performed a comparative biomarker study on patients with iNPH and subcortical small vessel disease(SSVD). In searching for pathophysiological similarities and differences, the authors selected biomarkers reflecting APP metabolism (subcortical damage and remodeling), neurofilament light protein (subcortical neural degeneration), glial fibrillary acidic protein (astroglial response), myelin basic protein (demyelination), and matrix metalloproteinases for subcortical tissue remodeling. By examining CSF biomarkers in patients with iNPH and SSVD, and in healthy controls (HC), they found similarities in the biomarker pattern of iNPH and SSVD, and differences with HC. This may indicate some common features in the underlying pathophysiology of iNPH and SSVD. The interrelation of these two pathological conditions might render iNPH “less idiopathic” and “more vascular” related.

Martinoni et al. prospectively evaluate an important differential diagnostic entity, LIAS, which may manifest with similar symptoms to iNPH but is treated primarily by endoscopic third ventriculostomy (ETV) instead of a shunt. A detailed anatomical visualization of the aqueduct with membrane identification by MRI is key for diagnosis. LIAS detection is important since ETV is more physiological than a shunt and avoids the mechanical complications of an implantable device. Postoperative neuro-cognitive improvement was seen mainly in attentive and executive functions, visuo-spatial memory, verbal executive functions, and behavioral and affective domains. With its pathophysiological insight, this study can help the understanding of some more common, broader mechanisms to iNPH CSF dynamics and contribute to differential diagnosis.

The majority of the papers directly investigate clinical diagnostic advancements and post-operative assessment. Clinical diagnosis refinement is the focus of articles by Kameda et al., Rydja et al., Wesner et al., and Kazui et al..

Kameda et al. attempt to improve the value of the tap test (TT) by refining the evaluation after it. For this purpose, they utilized the Functional Gait Assessment (FGA) and Global Rating of Change (GRC) scales. FGA and GRC demonstrated much higher sensitivity for TT predictive effectiveness than the 3-m Timed Up and Go test (TUG). It is found to be more justifiable to exclude FGA and GRC negative patients from surgery than the TUG negative patients.

Rydja et al. focus on postoperative motor function recovery in iNPH patients after shunting. They assess short-distance walking, functional exercise capacity, functional strength, and variables of activity and sleep, and evaluate the effect of a physical exercise program in the first 6 months after shunting. Both “exercise” and control groups improved at 3 and 6 months after shunting without significant differences between them. Remarkably, short-distance walking improvement is weakly correlated with voluntary walking, indicating that improved physical capacity does not directly translate to increased physical activity. Therefore, behavioral factors and participation of other frontal lobe mechanisms are important for motor recovery too.

Wesner et al. sought an empirically-based index to determine if iNPH patients show significant cognitive improvement after TT in a commonly used cognitive test, the Montreal Cognitive Assessment (MoCA). They compare various methods for estimating reliable change indices (RCIs) for MoCA in suspected iNPH patients undergoing TT or ELD. RCIs have already been applied as a strong empirically-based approach to improve clinical decision-making and cognitive changes in Parkinson's disease, stroke, and concussion patients (1). The study used estimated reliable change thresholds for MoCA in a population of older adults with suspected iNPH after the CSF drainage procedure (TT/ELD) and a subset of them with shunt surgery. The study obtains clinically applicable practical empirical standards for potential cognitive improvement following CSF drainage.

Kazui et al. explore ways for better cooperation between dementia specialists and neurosurgeons and evaluate, through a questionnaire, the network of specialized dementia institutions in Japan. They conclude that medical care for possible iNPH patients may be improved by dementia specialists performing CSF tap tests and sharing indications for shunt surgery with neurosurgeons. Certainly, better management of comorbidity in dementia patients can improve overall success.

Kikuta et al., Eide et al., and Keong et al. investigate an important current focus of iNPH research: patho-physiologically based imaging methods.

Kikuta et al. examine CSF diffusion along perivascular spaces, exploring the potential role of the glymphatic system in view of current pathophysiological theories. A number of studies show reduced perivascular flow in iNPH, and its improvement with CSF drainage. The authors explore the effect of CSF shunting (LPS) on this phenomenon. By applying the “Analysis along the perivascular space” index as an MRI technique, they compare patients before and after shunting, as well as responders and non-responders. Shunting and positive clinical response are shown to be associated with index increase, improved water diffusion, and better assumed glymphatic clearance.

Eide et al. perform a prospective observational study on 95 iNPH patients by evaluating recently introduced imaging biomarkers of CSF dynamics and glymphatic enhancement. They compare different intrathecal doses of gadobutrol (MRI contrast agent), aiming at the lowest reasonable, diagnostically informative dose. They find that tracer enrichment of subarachnoid CSF spaces (cisterna magna, vertex, and velum interpositum), ventricular reflux of tracer, and glymphatic tracer enrichment are dose-dependent. A reduction to 0.25 mmol gadobutrol concentration improves safety margins while remaining diagnostically informative on CSF homeostasis and glymphatic failure in iNPH.

Keong et al. attempted to reduce interpretation complexity of the diffusion tensor imaging (DTI) profiles of white matter disruption in hydrocephalic and non-hydrocephalic patients. The final investigated dataset was comprised of mild traumatic brain injury, NPH, and Alzheimer's disease patients vs. controls. By mapping tissue signatures included into the created “periodic table of DTI elements,” they rapidly characterized cohorts by their differing patterns of injury. The novel strategy with this “periodic table” interpretation allows distinguishing between the different cohorts along the spectrum of brain injury.

By paying attention to the effect of comorbidities, a multinational team (Goh et al.) from Malaysia, Singapore and the UK (Edinburgh and Oxford) evaluate clinical responses after ventriculo-peritoneal shunting in a cohort with coexisting NPH and neurodegenerative disease. They defined two categories patients with iNPH: Classic and Complex (Parkinson's, Alzheimer's diseases or vascular dementia comorbidity). They conducted follow ups with them for over one year and complete a retrospective pilot study and cohort analysis. They stratified the degree of comorbidity in the pilot study to criteria of probable or possible iNPH. After exclusions, 12 patients completed the pilot and 32 patients the retrospective study. The study concludes that the presence of neurodegenerative comorbidity should not preclude CSF tap/drainage tests, and further definition of comorbidity cohorts can provide better specialized treatment protocols for these patients.

Yamada et al., Kamo et al., and Kajimoto et al. investigate the outcome optimization of post-operative shunt management in iNPH patients.

Yamada et al. explore optimal pressure adjustment early and late after shunting. Residual symptoms may worsen again after several years, even when there is initial improvement after early optimal valve pressure setting. Because of the possibility of insufficient CSF drainage, valve pressure should be reduced by one step (2–4 cmH_2_O) 6 months to a year after shunting to maximize symptom improvement, followed by a head CT scan a month later.

Kamo et al. identify shunt malfunction with significant weight change and correct it with valve adjustment: in weight gain there was under-drainage and in weight loss, over-drainage. In the study, there were five cases of weight-related shunt malfunction with pressure environment assessment before, through and after the shunt malfunction, four cases of under drainage worsened gait disturbance with an average weight gain of 6.8 kg, and one over-drainage patient had an asymptomatic chronic subdural hematoma (CSDH) with a weight loss of 10 kg. For the weight-gain patients, intra-cranial and intra-abdominal pressures increased by 8.8 and 4.8 mmHg, respectively, and the ICP decrease was 5 mmHg for the weight-loss patient. Valve pressure re-adjustments led to the complete recovery of all patients, indicating the need for attention in patients with significant weight changes.

Kajimoto et al. investigate whether an early intervention has potential benefits and define a “prodromal” iNPH stage (TUG ≤ 13.5 s and MMSE ≥ 24). They evaluate 12 such cases over 4 years from the pool of treated patients at their institution with the iNPH Grading Scale, Frontal Assessment Battery, intermittent gait disturbances, social participation status, and development of comorbidities. Early intervention in the prodromal iNPH stage maintained good cognition, mobility, and social participation ability in the long term. The maintenance of long-term cognitive function suggests a preventive effect on dementia. Early intervention for iNPH will require the application of earlier diagnostic protocols.

The Research Topic is complemented by the mini-review of Langheinrich et al. on cognitive presentations of iNPH for clinicians, a useful adjunct for clinical practice.

The present collection is the next step toward a better understanding of iNPH. We are still seeking to establish the specific pathophysiology of this condition with the co-existing structural changes at all levels, from molecular to macroscopic, and the continuity of such diverse exploration of iNPH will be crucial for achieving a breakthrough on this subject.

## Author contributions

All authors listed have made a substantial, direct, and intellectual contribution to the work and approved it for publication.

## Conflict of interest

The authors declare that the research was conducted in the absence of any commercial or financial relationships that could be construed as a potential conflict of interest.

## Publisher's note

All claims expressed in this article are solely those of the authors and do not necessarily represent those of their affiliated organizations, or those of the publisher, the editors and the reviewers. Any product that may be evaluated in this article, or claim that may be made by its manufacturer, is not guaranteed or endorsed by the publisher.
